# Mindfulness as a Protective Factor Against Orthorexia: The Mediating Role of Body Image Anxiety

**DOI:** 10.3390/bs16050665

**Published:** 2026-04-28

**Authors:** Mirsini Pappa, Ioanna Christina Kostoula, Efstratios Christodoulou, Georgia-Eirini Deligiannidou, Antonios E. Koutelidakis, Theodoros Konstantinidis, Christos Kontogiorgis

**Affiliations:** 1 Laboratory of Nutritional and Public Health, Department of Food Science and Nutrition, University of the Aegean, 81400 Myrina, Greece; mirt.pap@hotmail.com (M.P.); akoutel@aegean.gr (A.E.K.); 2 Laboratory of Hygiene and Environmental Protection, School of Medicine, Democritus University of Thrace, Dragana, 68100 Alexandroupolis, Greece; i.c.kostoula@gmail.com (I.C.K.); edeligia@med.duth.gr (G.-E.D.); tconstan@med.duth.gr (T.K.)

**Keywords:** mindfulness, body image, orthorexia, eating behavior, mediation

## Abstract

Mindfulness and body image anxiety are psychological factors associated with disordered eating and may contribute to orthorexia nervosa, yet their combined effects remain underexplored. In this cross-sectional online survey, 382 adults in Greece completed the Mindful Attention Awareness Scale (MAAS-15), the Orthorexia scale (ORTO-6), the Social Physique Anxiety Scale (SPAS-12), and measures of body mass index (BMI) and physical activity (PAVS). Descriptive statistics, correlations, regression analyses, and mediation analysis were conducted to examine the associations among mindfulness, body image anxiety, and orthorexia. Mindfulness correlated negatively with orthorexia and body image anxiety, whereas body image anxiety correlated positively with orthorexia. In multinomial logistic regression, higher body image anxiety increased the odds of low (OR = 1.194, 95% CI 1.114–1.280) and moderate mindfulness (OR = 1.125, 95% CI 1.068–1.185); orthorexia also increased the odds of low (OR = 1.146, 95% CI 1.040–1.264) and moderate mindfulness (OR = 1.099, 95% CI 1.026–1.176). Overall, psychological factors (mindfulness, body image anxiety) appeared more influential than anthropometric or lifestyle factors (BMI, physical activity) in relation to orthorexia. These findings indicate that mindfulness was inversely associated with orthorexia tendencies, while body image anxiety was positively associated with orthorexia and was statistically linked to this association in the mediation analysis.

## 1. Introduction

In recent years, mindfulness has emerged as a central psychological construct associated with mental well-being and adaptive self-regulation. Defined as a non-judgmental awareness of the present moment (Brown & Ryan, 2003), mindfulness fosters cognitive flexibility and emotional balance, enabling individuals to disengage from automatic, rigid patterns of thinking and behavior. Within the domain of eating behaviors, mindfulness has been linked to healthier dietary patterns, reduced emotional eating, and greater body acceptance (Kristeller & Wolever, 2010; Sala et al., 2020).

Orthorexia nervosa (ON), a term first introduced by Bratman (Dunn & Bratman, 2016), describes a pathological fixation with consuming only “pure,” “healthy,” or “clean” foods. Although originally motivated by health concerns, this rigid adherence to perceived healthy eating can result in psychological distress, social isolation, and nutritional deficiencies (Cena et al., 2019; Dunn & Bratman, 2016). While ON is not officially recognized in diagnostic manuals (e.g., DSM-5), empirical research has increasingly identified it as a distinct disordered eating pattern characterized by obsessive tendencies, perfectionism, and cognitive inflexibility (Lee et al., 2024).

Recent reviews indicate that orthorexia nervosa remains an evolving construct, with ongoing debate regarding its prevalence, diagnostic boundaries, and measurement, while evidence increasingly supports its clinical relevance in relation to psychological distress, functional impairment, and overlap with other eating-disorder features (Lee et al., 2024). The literature suggests that orthorexia should not be viewed as a unitary construct, as a growing body of research distinguishes orthorexia nervosa from healthy orthorexia. Whereas orthorexia nervosa reflects a rigid and impairing preoccupation with healthy eating, healthy orthorexia refers to a more adaptive, non-pathological interest in food quality and healthy nutrition. This distinction is conceptually important because it helps avoid over-pathologizing health-conscious eating and supports a more nuanced interpretation of orthorexia-related tendencies (Brytek-Matera et al., 2015; Barlow et al., 2024).

From a theoretical perspective, mindfulness may be associated with self-regulation and acceptance-related processes that may partly account for its relationship with body-related anxieties. According to self-regulation and Acceptance and Commitment Theory (Hart et al., 1989), mindfulness has been described as involving greater awareness and acceptance of internal experiences, thereby reducing the tendency to over-control or avoid emotions through rigid food rules. By cultivating non-judgmental awareness, mindfulness may offer one possible framework for understanding the observed association between body image anxiety and orthorexia tendencies.

Despite growing evidence that mindfulness is inversely related to disordered eating, limited attention has been paid to the mechanisms explaining this relationship, particularly the mediating role of body image anxiety. Most studies have examined mindfulness and orthorexia independently, neglecting how body image concerns might function as an explanatory bridge.

This study aimed to explore the associations among mindfulness, body image anxiety, and orthorexia nervosa in a sample of Greek adults, with a particular focus on the mediating role of body image anxiety.

Hypotheses:

1. Mindfulness will be negatively associated with orthorexia.
2. Body image anxiety will be positively associated with orthorexia.
3. Body image anxiety will be statistically associated with the relationship between mindfulness and orthorexia tendencies.

## 2. Materials and Methods

### 2.1. Study Design and Procedure

The present study employed a cross-sectional online survey design. Data were collected using the SoGoSurvey (Sogolytics) platform, which ensures secure, anonymous, and GDPR-compliant data management. The questionnaire was available in Greek and included demographic questions along with validated Greek versions of the psychometric instruments assessing mindfulness (MAAS-15) (Gopan et al., 2023) orthorexia tendencies (ORTO-6), body image anxiety (SPAS-12) (Hart et al., 1989), body mass index (BMI) and physical activity (PAVS) (Lin et al., 2022). Participants were eligible if they were aged 18 to 65 years, resided in Greece, and completed the survey. No formal exclusion criteria were applied regarding current eating disorder or other psychiatric diagnoses.

The survey link was disseminated through convenience and snowball sampling via the authors’ personal and professional networks, including Facebook, Instagram, LinkedIn, and email. Given the online and exploratory nature of the study, this non-probability sampling approach was used to recruit a broad community sample of Greek-speaking adults residing in Greece.

Data collection was conducted over a two-month period (June–August 2025). The SoGoSurvey platform automatically restricts multiple submissions from the same IP address to ensure data integrity and uniqueness.

A pilot study was conducted with the first 50 responses to ensure the clarity and functionality of the questionnaire. Since no significant issues were identified, we proceeded with the main data collection phase, retaining the responses from the pilot study as part of the final sample.

The study was conducted in a non-clinical community sample and did not require a diagnosis of orthorexia nervosa as an inclusion criterion. Instead, orthorexia tendencies were assessed dimensionally using the ORTO-6.

### 2.2. Ethics Statement

This study was conducted in accordance with the Declaration of Helsinki and approved by the Ethics Committee of the University of the Aegean (Protocol code: 2025/07). Participation was voluntary, and respondents could withdraw at any stage without penalty. No identifying information was collected. The study did not receive external funding, and the authors declare no conflicts of interest.

### 2.3. Measures

Mindfulness

Mindfulness was measured using the Mindful Attention Awareness Scale (MAAS-15) developed by Brown and Ryan (Brown & Ryan, 2003). This scale consists of 15 items assessing dispositional mindfulness, rated on a 5-point Likert scale (1 = almost always to 5 = almost never). Higher scores indicate greater mindfulness.

Orthorexia Nervosa

Orthorexia tendencies were assessed using the ORTO-6 (Missbach et al., 2015), a brief version of the original ORTO-15 adapted for improved psychometric performance. The ORTO-6 consists of six items; each rated on a 4-point Likert scale.

Body Image Anxiety

Body image anxiety was measured with the Social Physique Anxiety Scale—12 item version (SPAS-12) (Hart et al., 1989). The SPAS-12 assesses anxiety related to the perception of one’s body by others, rated on a 5-point Likert scale (1 = strongly disagree to 5 = strongly agree). Higher scores denote greater body image anxiety.

Body Mass Index (BMI)

BMI was calculated from self-reported weight and height (kg/m^2^). Although self-reported measures may introduce bias, they are widely used and reliable in online epidemiological studies (Pursey et al., 2014).

Physical Activity (PAVS)

The Physical Activity Vital Sign (PAVS) (Blair et al., 1985; Greenwood et al., 2010) was used to estimate participants’ weekly physical activity. The PAVS comprises two questions assessing the number of days per week and average minutes per day spent in moderate-to-vigorous physical activity. Total minutes per week were calculated by multiplying these two responses.

## 3. Results

### 3.1. Participants and Demographic Characteristics

The final sample consisted of 382 eligible participants (out of 397 total responses) after data screening. The mean age of participants was 33.9 years (SD = 9.1), with most respondents falling within the 18–29 years age group (49%), followed by those aged 30–39 years (27%), 40–49 years (13.4%), and 50–65 years (10.7%). Regarding gender, the majority identified as female (64.3%), while 34.9% identified as male, and a small proportion (0.8%) preferred not to disclose their gender. The mean Body Mass Index (BMI) of the sample was 25.24 kg/m^2^ (SD = 4.89). Approximately 43.5% of participants had a BMI within the normal range (18.5–24.9 kg/m^2^), 36.9% were overweight (25–29.9 kg/m^2^), 16.3% were classified as obese (≥30 kg/m^2^), and 3.1% as underweight (<18.5 kg/m^2^). These characteristics are presented in Table 1.

### 3.2. Descriptive Data and Reliability Summary

Summary statistics and internal consistency coefficients for the main study variables are presented in Table 2. Cronbach’s alpha was 0.888 for MAAS-15, 0.681 for ORTO-6, and 0.743 for SPAS-12, indicating acceptable to good internal consistency. The mean mindfulness score was 3.77 (SD = 0.87), reflecting the average mindfulness score observed in the sample. The mean BMI was 25.24 (SD = 4.89), while the mean PAVS score was 152.05 min/week (SD = 168.88) in the study sample. The mean SPAS-12 score was 33.30 (SD = 5.51), and the mean ORTO-6 score was 18.42 (SD = 3.89). Overall, the descriptive findings indicate variability across the main psychological and behavioral variables examined in the study.

Spearman’s correlation analysis revealed a significant negative correlation between mindfulness (MAAS-15) and orthorexia tendencies (ORTO-6), ρ = −0.316, *p* < 0.001. Higher mindfulness scores were associated with lower orthorexia tendencies in this sample.

A multiple regression model also indicated a significant association between mindfulness and orthorexia tendencies. When orthorexia was predicted from mindfulness, body image anxiety, and BMI, the model explained 21.9% of the variance, F(3,378) = 35.31, *p* < 0.001. Mindfulness emerged as a significant negative predictor of orthorexia (B = −0.811, β = −0.180, *p* < 0.001), even when controlling for other variables (Table 3).

These results indicate that greater dispositional mindfulness was associated with lower orthorexia tendencies in this sample. Psychological variables, particularly mindfulness and body image anxiety, accounted for more variance in orthorexia tendencies than BMI.

### 3.3. Mindfulness and Body Image Anxiety

Mindfulness was negatively associated with body image anxiety (SPAS-12), ρ = −0.378, *p* < 0.001. This moderate-to-strong relationship suggests that greater mindfulness relates to less concern and distress about body shape and appearance.

A regression model predicting body image anxiety from mindfulness and orthorexia was significant, F(2,379) = 71.89, *p* < 0.001, explaining 27.5% of the variance (R^2^ = 0.275). Both predictors were significant: mindfulness negatively (B = −1.950, β = −0.307, *p* < 0.001), and orthorexia positively (B = 0.474, β = 0.335, *p* < 0.001) (Table 4).

Individuals with higher mindfulness report less anxiety about their physical appearance, even when orthorexia tendencies are accounted for. Mindfulness may help individuals observe their thoughts about their body with greater acceptance, reducing pressure to meet unrealistic body ideals.

Body Image Anxiety and Orthorexia: A Risk Pathway

Body image anxiety (SPAS-12) was positively correlated with orthorexia (ORTO-6), ρ = 0.410, *p* < 0.001, indicating that individuals who worry more about their appearance also exhibit stronger tendencies toward orthorexia behaviors.

In the multiple regression model predicting orthorexia, body image anxiety was the strongest predictor (B = 0.256, β = 0.362, *p* < 0.001). Higher body image anxiety was associated with higher orthorexia tendencies in this sample. This pattern is consistent with a positive association between body image anxiety and orthorexia tendencies.

The Mediating Role of Body Image Anxiety

To examine whether body image anxiety explains part of the relationship between mindfulness and orthorexia, a mediation analysis following Baron and Kenny’s steps was performed. The results of the mediation analysis are presented in Table 5.

**Figure 1:**
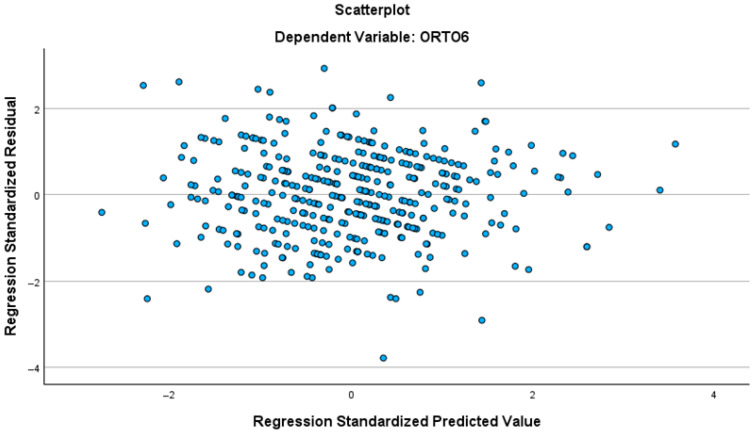
Association between mindfulness and orthorexia tendencies, with body image anxiety showing a partial indirect association.

The coefficient for mindfulness decreased from −1.497 to −0.817 after the inclusion of body image anxiety, while remaining statistically significant. This pattern is consistent with a partial indirect association involving body image anxiety. These findings suggest that the association between mindfulness and orthorexia tendencies may be partly statistically linked to body image anxiety, as illustrated in Figure 1.

Secondary Findings: BMI and Physical Activity

Although the primary focus of this study was the interplay between mindfulness, body image anxiety, and orthorexia, additional analyses were conducted to explore the contribution of BMI and physical activity (PAVS) to mindfulness categories. A multinomial logistic regression was used to identify predictors differentiating low and moderate mindfulness levels (Categories 1 and 2) from high mindfulness (reference Category 3).

**Figure 2:**
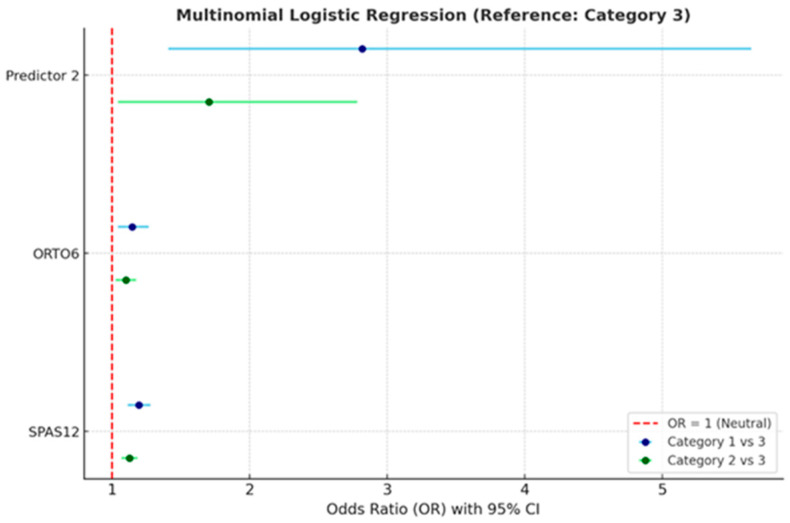
Forest plot of multinomial logistic regression results with Category 3 (High Mindfulness) as the reference.

The model included body image anxiety (SPAS-12), orthorexia tendencies (ORTO-6), and an additional categorical predictor (“Predictor 2,” e.g., gender or BMI group). Results are summarized in Table 6 and illustrated in Figure 2.

All significant predictors SPAS-12, ORTO-6, and Predictor 2 showed odds ratios greater than 1, indicating that higher levels of these variables increase the likelihood of belonging to lower mindfulness categories (1 or 2) compared to the reference (3).

Specifically, body image anxiety exerted the strongest influence: each one-point increase in SPAS-12 raised the odds of falling into Category 1 (low mindfulness) by 19.4% and into Category 2 (moderate mindfulness) by 12.5%. Similarly, orthorexia tendencies also elevated the risk of lower mindfulness by 14.6% for Category 1 and 9.9% for Category 2. Finally, Predictor 2 showed a strong association with Category 1 (OR = 2.818), suggesting that certain demographic or lifestyle factors may also affect mindfulness levels.

**Figure 3:**
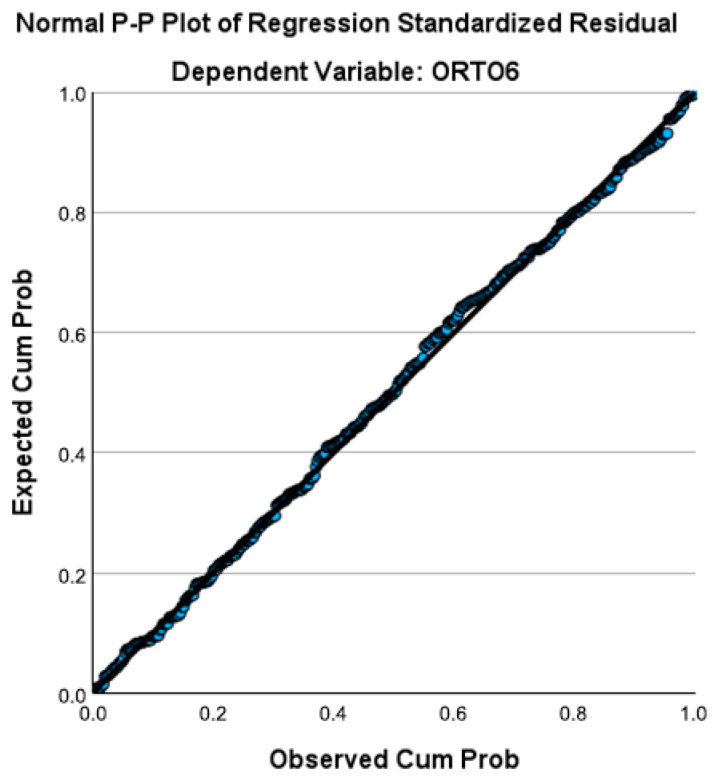
Forest plot of multinomial logistic regression results with Category 3 as the reference.

Odds ratios (OR) with 95% confidence intervals (CI) are shown in Figure 3 for body image anxiety (SPAS-12), orthorexia (ORTO-6), and the additional predictor (Predictor 2). Blue markers represent Category 1 vs. 3 comparisons, while green markers correspond to Category 2 vs. 3. The red dashed line at OR = 1 indicates the null value. OR values > 1 with CIs not crossing 1 indicate statistically significant increased likelihood of belonging to the respective lower mindfulness category.

**Figure 4:**
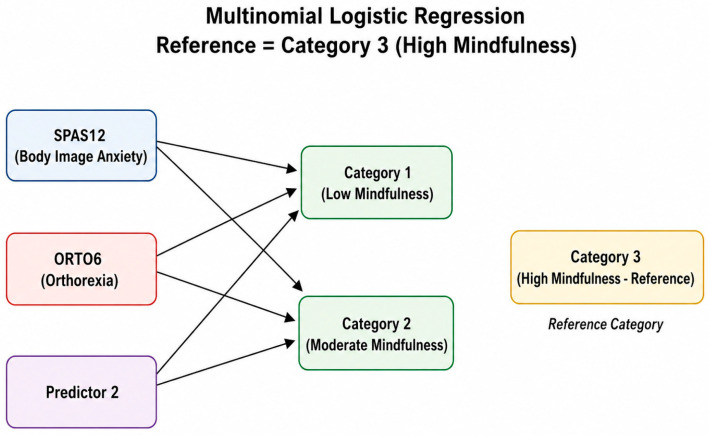
Conceptual map of predictors associated with mindfulness categories.

The conceptual model of predictors associated with mindfulness categories is illustrated in Figure 4.

This schematic representation illustrates how higher body image anxiety (SPAS-12) and orthorexia tendencies (ORTO-6) are linked with lower mindfulness levels (Categories 1 and 2) compared to the reference group (Category 3—high mindfulness).

Arrows indicate the direction of associations, emphasizing that increases in these psychological variables correspond to a greater likelihood of belonging to lower mindfulness categories. “Predictor 2” denotes an additional demographic or categorical factor (e.g., BMI group or gender) that also contributes to mindfulness variation.

These findings suggest that psychological factors, particularly body image anxiety and orthorexia tendencies, play a substantial role in determining mindfulness levels.

Individuals with higher anxiety about body image and stronger preoccupation with healthy eating were significantly more likely to exhibit lower mindfulness. This pattern may reflect that self-critical or perfectionistic thinking, common in both body dissatisfaction and orthorexia, undermines one’s ability to remain nonjudgmentally aware of the present moment.

By contrast, BMI and physical activity did not emerge as significant contributors, reinforcing that psychological rather than anthropometric variables explain most of the differences in mindfulness.

**Figure 5:**
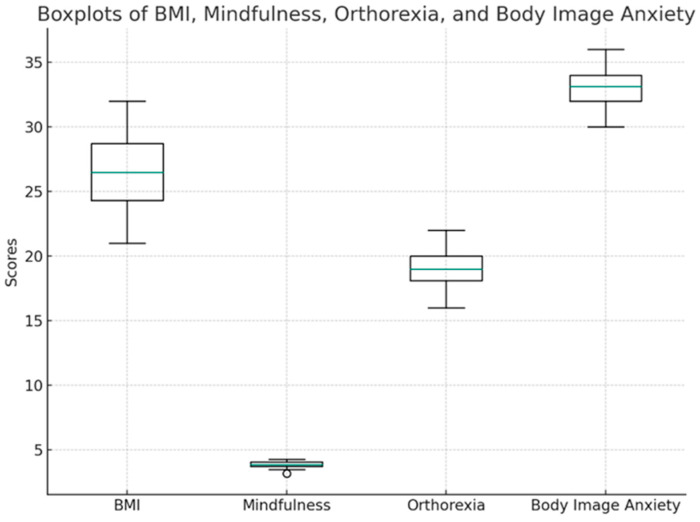
Boxplots of BMI, mindfulness, orthorexia, and body image anxiety.

In practical terms, this means that even individuals with similar body weights or exercise habits may differ in mindfulness depending on their internal cognitive–emotional patterns—such as anxiety about appearance or rigid attitudes toward food. Hence, mindfulness interventions could be especially useful for reducing self-critical and appearance-focused thinking, which appears to erode mindful awareness. The distributions of BMI, mindfulness, orthorexia, and body image anxiety are presented in Figure 5.

**Figure 6:**
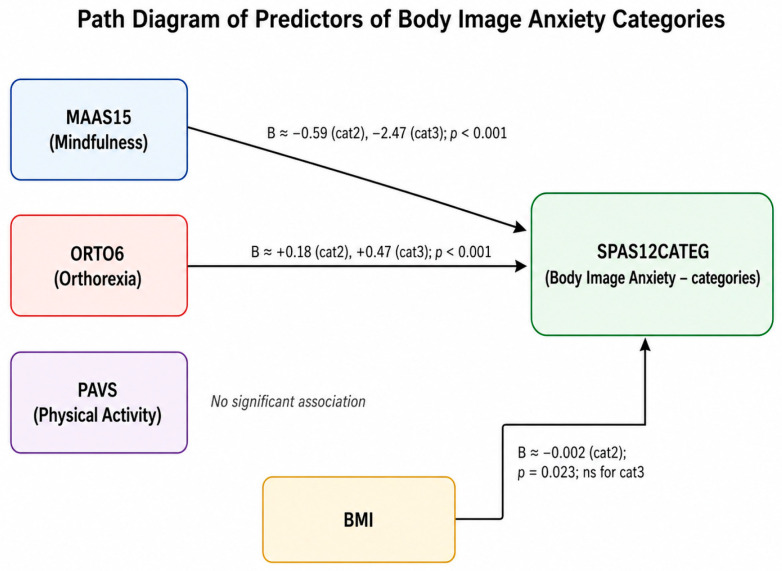
Path diagram of predictors of body image anxiety categories (SPAS12CATEG).

The figure displays the distribution, median, and interquartile range for each variable. BMI shows moderate variability, with a median around 25. Mindfulness scores are tightly clustered around 3.8–4.0, orthorexia around 18–19, and body image anxiety around 33–34, reflecting elevated but consistent levels across participants. The boxplots indicate that all variables are approximately symmetrically distributed, with a few mild outliers. The predictors of body image anxiety categories are illustrated in Figure 6.

Overall, the figure visually confirms that the dataset is balanced and that no extreme outliers distort the results. It provides a clear snapshot of how participants scored across the major constructions.

## 4. Discussion

The present study examined the associations among mindfulness, body image anxiety, and orthorexia tendencies. Mindfulness was negatively associated with both orthorexia and body image anxiety, whereas body image anxiety was positively associated with orthorexia. In addition, the mediation analysis indicated that body image anxiety was statistically linked to the association between mindfulness and orthorexia within the proposed mode.

These findings align with previous studies reporting associations between mindfulness and lower levels of disordered eating-related symptoms and body-related distress (Lee et al., 2024; Kwan et al., 2018). In the present study, higher self-reported mindfulness was associated with lower orthorexia tendencies and lower body image anxiety (Kristeller & Wolever, 2010). This pattern is also consistent with recent findings showing negative associations of orthorexia nervosa with mindfulness, mindful eating, and self-compassion in non-clinical samples (Kalika et al., 2023). In contrast, those with elevated body image anxiety often display maladaptive attempts to manage perceived flaws through strict dietary restraint or avoidance of “unhealthy” food (Brytek-Matera et al., 2015; Gopan et al., 2023; Barlow et al., 2024).

In addition, recent meta-analytic evidence suggests that mindfulness-based interventions may be associated with improvements in body image dissatisfaction, further supporting the broader relevance of mindfulness-related processes in body-related psychological functioning. Although the present study did not examine intervention effects, this evidence provides useful context for interpreting the observed association between mindfulness and body image anxiety (Gopan et al., 2023).

The present findings should also be interpreted in light of the growing distinction between healthy orthorexia and orthorexia nervosa. While the current study assessed orthorexia tendencies using the ORTO-6, the recent literature suggests that health-focused eating may include both adaptive and maladaptive dimensions. In this context, the observed associations of orthorexia tendencies with body image anxiety and lower mindfulness appear more consistent with the pathological features typically attributed to orthorexia nervosa than with a non-problematic interest in healthy eating. At the same time, this conceptual distinction highlights the need for caution when interpreting orthorexia-related scores, particularly given ongoing concerns regarding the measurement consistency of some orthorexia instruments, including ORTO-based tools. Future studies would benefit from using measures that explicitly distinguish healthy orthorexia from orthorexia nervosa (Cena et al., 2019).

From a theoretical perspective, these results can be interpreted within self-regulation and acceptance frameworks (Baumeister et al., 2007). As individuals become more attuned to their experiences without judgment, they may be less susceptible to external ideals of “purity” or appearance-based worth. This mechanism resonates with Acceptance and Commitment Theory (ACT), which emphasizes psychological flexibility and value-based behavior rather than control-oriented coping. Therefore, these frameworks may offer one possible context for understanding the observed association among mindfulness, body image anxiety, and orthorexia tendencies.

Another key finding is that psychological variables (mindfulness and body image anxiety) were stronger predictors of orthorexia than anthropometric indicators (BMI) or lifestyle factors (physical activity). These findings indicate that orthorexia tendencies were more strongly associated with psychological than anthropometric or lifestyle-related variables (Dunn & Bratman, 2016; Barlow et al., 2024).

The results also contribute to the growing literature identifying body image anxiety as a transdiagnostic factor across eating and obsessive–compulsive spectrum disorders (Minari et al., 2024). Interventions that target body-related anxiety, combined with mindfulness-based strategies, may be particularly effective in addressing orthorexia tendencies. Programs such as Mindfulness-Based Eating Awareness Training (MB-EAT) or Mindfulness-Based Cognitive Therapy (MBCT) have shown promising results in reducing preoccupation with food and improving body satisfaction (Kristeller & Wolever, 2010).

### Study Limitations

This study has several limitations that should be acknowledged. First, its cross-sectional design does not permit causal inferences regarding the relationships among mindfulness, body image anxiety, and orthorexia tendencies. Second, all data were collected through self-reported measures, which may be subject to recall and social desirability bias. Third, the sample was recruited through convenience and snowball sampling via online platforms, which limits the representativeness of the sample and the generalizability of the findings to the broader Greek population. This sampling strategy may also have introduced selection bias, as individuals with greater interest in nutrition, mental health, or body-related concerns may have been more likely to participate. Moreover, recruitment through an online survey may have excluded individuals with limited internet access or lower digital literacy. Data collection took place between June and August 2025, and seasonal or contextual factors related to this period may have influenced eating behaviors, body-related perceptions, or physical activity patterns. In addition, no specific information was collected regarding current or past diagnoses of eating disorders or other psychiatric conditions, which may represent a potential confounding factor.

The study also did not recruit participants on the basis of a clinical diagnosis of orthorexia nervosa. As a result, the proportion of individuals with elevated orthorexia tendencies may have been relatively small, which limits the extent to which the findings can be generalized to clinical populations. Taken together, these limitations are particularly important when interpreting the mediation findings, which should be understood as cross-sectional statistical associations rather than evidence of temporal or causal pathways.


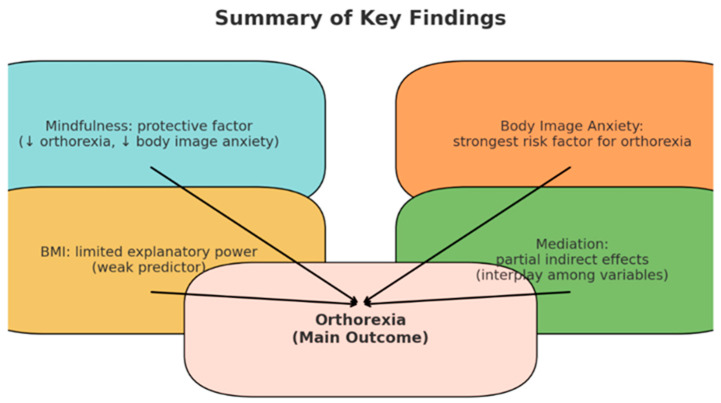


## 5. Conclusions

Overall, the findings indicate that mindfulness was inversely associated with orthorexia tendencies, while body image anxiety was statistically linked to this association within the proposed mediation model.

## Figures and Tables

**Table 1:** Demographic Characteristics and Distribution of the Study Sample.

Demographic Item	Male *N* (%)	Female *N* (%)	Other *N* (%)	Total *N* (%)	*p*-Value *
*Age groups (years)*					
18–29	67 (49.6)	118 (48.2)	2 (66.7)	187 (49.0)	—
30–39	36 (26.7)	66 (27.0)	1 (33.3)	103 (27.0)	—
40–49	17 (12.6)	34 (13.9)	0 (0.0)	51 (13.4)	—
50–65	15 (11.1)	26 (10.6)	0 (0.0)	41 (10.7)	—
Total (Valid)	135 (100.0)	244 (100.0)	3 (100.0)	382 (100.0)	—
*Age*					
Mean ± SD	33.8 ± 9.5	34.2 ± 9.0	—	33.9 ± 9.1	0.84
Median	32	33	—	33	—
*BMI Classes (kg/m^2^)*					
<18.5	4 (3.0)	8 (3.3)	0 (0.0)	12 (3.1)	—
18.5–24.9	57 (42.2)	107 (43.9)	2 (66.7)	166 (43.5)	—
25–29.9	50 (37.0)	90 (36.9)	1 (33.3)	141 (36.9)	—
≥30	24 (17.8)	38 (15.6)	0 (0.0)	62 (16.3)	—
BMI (Mean ± SD)	25.36 ± 4.97	25.18 ± 4.83	—	25.24 ± 4.89	0.74
Median	25.0	24.9	—	25.0	—

* *p*-values refer to comparisons between male and female participants.

**Table 2:** Descriptive Statistics of Main Variables.

Variable	Male *N*	Female *N*	Male Mean (SD)	Female Mean (SD)	Total Mean (SD)	Cronbach’s α
Mindfulness (MAAS-15)	133	245	3.82 (0.86)	3.75 (0.88)	3.77 (0.87)	0.86
BMI	133	245	25.36 (4.97)	25.18 (4.83)	25.24 (4.89)	—
Physical Activity (PAVS)	133	245	170.3 (160.2)	152.5 (168.9)	158.0 (165.5)	0.77
Body Image Anxiety (SPAS-12)	133	245	34.1 (10.5)	37.8 (11.4)	36.4 (11.2)	0.88
Orthorexia (ORTO-6)	133	245	21.7 (4.1)	22.0 (4.3)	21.9 (4.2)	0.81

**Table 3:** Multiple Regression Analysis Predicting Orthorexia Tendencies.

Predictor	B	β	t	*p*
Mindfulness (MAAS-15)	−0.811	−0.180	−3.59	<0.001
Body Image Anxiety (SPAS-12)	0.256	0.362	7.23	<0.001
BMI	−0.000004	−0.014	−0.31	0.761

**Table 4:** Regression Model of Orthorexia and Mindfulness as Predictors of Body Image Anxiety.

Predictor	B	SE	β	t	*p*
Orthorexia (ORTO-6)	0.474	0.066	0.335	7.23	<0.001
Mindfulness (MAAS-15)	−1.950	0.295	−0.307	−6.61	<0.001

**Table 5:** Mediation Analysis Examining the Role of Body Image Anxiety in the Relationship Between Mindfulness and Orthorexia.

Path	B	β	t	*p*
c: Mindfulness → Orthorexia	−1.497	−0.333	−7.09	<0.001
a: Mindfulness → Body Image Anxiety	−2.660	−0.418	−9.52	<0.001
b: Body Image Anxiety → Orthorexia (controlling MAAS)	0.255	0.361	7.23	<0.001
c′: Mindfulness → Orthorexia (controlling SPAS)	−0.817	−0.182	−3.59	<0.001

Note. c = total effect of mindfulness on orthorexia; a = effect of mindfulness on body image anxiety; b = effect of body image anxiety on orthorexia controlling for mindfulness; c′ = direct effect of mindfulness on orthorexia controlling for body image anxiety.

**Table 6:** Multinomial Logistic Regression Predicting Lower Mindfulness Categories.

Predictors	B	S.E.	Wald	*p*	OR = Exp (B)	95% CI (OR)
Category 1 vs. 3						
SPAS-12	0.177	0.035	25.081	<0.001	1.194	1.114–1.280
ORTO-6	0.137	0.050	7.503	0.006	1.146	1.040–1.264
Predictor “2”	1.036	0.354	8.549	0.003	2.818	1.407–5.645
Category 2 vs. 3						
SPAS-12	0.118	0.026	19.738	<0.001	1.125	1.068–1.185
ORTO-6	0.094	0.035	7.252	0.007	1.099	1.026–1.176
Predictor “2”	0.532	0.251	4.515	0.034	1.703	1.042–2.783

## Data Availability

The data supporting the findings of this study are available from the corresponding author upon reasonable request.
